# Analysis of 18 mercapturic acids in urine samples from the German Environmental Specimen Bank—tackling the data gap in the human biomonitoring of VOCs in Europe

**DOI:** 10.1038/s41370-026-00838-x

**Published:** 2026-01-28

**Authors:** Nikola Pluym, Therese Burkhardt, Till Weber, Gerhard Scherer, Max Scherer, Marike Kolossa-Gehring

**Affiliations:** 1 ABF Analytisch-Biologisches Forschungslabor GmbH, Planegg, Germany; 2 German Environment Agency (UBA), Berlin, Germany

**Keywords:** VOC, Human Biomonitoring (HBM), Time trend, Environmental Specimen Bank, Exposure assessment, Mercapturic acid

## Abstract

**Background:**

Human biomonitoring (HBM) plays a pivotal role in assessing exposure to toxicologically relevant chemicals, with urinary metabolites serving as key indicators. Despite the widespread implementation of HBM programs globally, certain metabolites, such as mercapturic acids (MAs) derived from volatile organic compounds (VOCs), remain understudied in non-occupationally exposed populations.

**Objective:**

To bridge this data gap, we analyzed 18 MAs in 360 24-h urine samples collected over a time span of 21 years from 2000 to 2021 in Germany.

**Methods:**

Two LC-MS/MS methods were utilized to quantify MAs in urine samples obtained from the Environmental Specimen Bank. Statistical analyses, including Kruskal-Wallis tests and Spearman correlations, were employed to evaluate temporal trends, sex-specific differences, and correlations between MAs.

**Results:**

Quantification rates between 95 and 100% were obtained for 14 of the 18 MAs, with notable variations in concentrations among different metabolites. The most pronounced decrease in MA levels was observed from 2010/2015 to 2019 with a significant trend for 8 MAs, potentially reflecting changes in environmental exposures and regulations. Moreover, significant differences in urinary excretion per 24 h between males and females were observed for several MAs, highlighting the importance of considering sex in exposure assessments.

**Impact Statement:**

Comprehensive human biomonitoring (HBM) data with regard to the exposure to volatile organic compounds (VOCs) in Europe is lacking. This prompted us to quantify 18 mercapturic acids as urinary VOC metabolites in 360 samples from the Environmental Specimen Bank collected between 2000 and 2021 in Germany. Our study demonstrates the ubiquitous exposure to numerous VOCs of high toxicological relevance, albeit with a decreasing trend over time for most of the metabolites. This emphasizes the need for a broader HBM to better understand the risk of VOC exposure in Germany and more general in Europe.

## Introduction

Human biomonitoring (HBM) deals with the assessment of internal exposure to toxicologically relevant chemicals by measuring them and/ or their specific metabolites in biospecimens such as blood or urine. The growing importance of HBM in the chemical risk assessment is underscored by its integration into the Green Deal as part of the European chemicals strategy towards a toxic free environment. HBM programs have been launched in several countries for decades inter alia as part of extensive health surveys in population-representative samples, including the National Health and Nutrition Examination Survey in the US (NHANES) [1] as the most comprehensive program worldwide, the German Environmental Survey (GerES) [2], Korea National Health and Nutrition Examination Survey (KNHANES) [3] or the Canadian Health Measures Survey (CHMS) [4]. As a major part of these surveys, several environmental chemicals and their metabolites have been periodically monitored to observe changes in the exposure over time, and to identify causal relationships with the individual behavior, environmental factors, and regional differences. Besides Germany several European countries have established national surveys such as FLEHS in Belgium [5], the Czech Republic’s HBM program (CZ-HBM) [6] and the French national HBM program [7] and biobanks for the analyses of time trends like the German Environmental Specimen Bank [8]. While these HBM programs have a similar scope, there are variations in recruitment, sample collection, analytical measurement, and data evaluation strategies. Recognizing the need for harmonization at the European level and addressing data gaps for previously overlooked toxicologically relevant chemicals, a joint European program co-funded by the European Commission—The European Human Biomonitoring Initiative (HBM4EU)—was launched in 2017. The initiative ran for five and a half years focusing on harmonizing HBM practices [9, 10], collecting new Europewide HBM data, and bridging data gaps for specific toxicants [11, 12].

This paper addresses the limited assessment of mercapturic acids (MAs), which represent the urinary elimination products of volatile organic compounds (VOCs). VOCs (e.g., benzene, toluene, acrylonitrile, acrolein, 1,3-butadiene) are electrophiles themselves or form electrophilic intermediates during metabolism. As a result, they can readily react with nucleophilic sites of physiological macromolecules such as DNA, RNA or proteins, which explains their toxic potential. The main detoxification process encompasses the addition of the electrophiles to glutathione and subsequent excretion as mercapturic acids [13], which can be determined in urine as biomarkers of VOC exposure. While NHANES routinely quantifies 24 MAs covering a broad spectrum of VOCs [14], European, national HBM programs have infrequently examined MAs to date.

In Germany, the Environmental Specimen Bank (ESB) complements GerES by storing, among others, urine samples over liquid nitrogen to retrospectively assess chemical exposures over time [15]. The cryostorage coupled with detailed documentation of sex, age, and 24 h urine volume allows for a comprehensive elucidation of time series and sex-specific differences in exposure assessment [8]. In this study, 18 MAs derived from 14 VOCs were quantified in 360 24-h urine samples from the ESB over a 21-year period from 2000 to 2021. Structures of the MAs along with their corresponding precursor chemicals are summarized in Table 1. The aim was to fill existing data gaps on toxicant exposure in Europe, in accordance with the scope of HBM4EU.

**Table 1:** Names, abbreviations and structures of the mercapturic acids analyzed and their corresponding precursor chemicals.

Name	Abbr.	Chemical structure	Name of precursor	Structure of precursor	IARC group / REACH classification
*N*-Acetyl-*S*-methyl-L-cysteine	MMA		methylating agents, e.g., NDMA, methyl chloride		REACH: Suspected to be carcinogenic (methyl chloride)
*N*-Acetyl-*S*-ethyl-L-cysteine	EMA		ethylating agents, e.g., NDEA, ethyl chloride		IARC group 3 (ethyl chloride); REACH: Suspected to be carcinogenic (ethyl chloride)
*N*-Acetyl-*S*-(2-hydroxyethyl)-L-cysteine	2HEMA		ethylene oxide, acrylonitrile		REACH: carcinogenic, mutagenic, toxic to reproduction (ethylene oxide); IARC group 1 (acrylonitrile); REACH: carcinogenic, skin sensitizing (acrylonitrile)
*N*-Acetyl-*S*-(2-hydroxypropyl)-L-cysteine	2HPMA		propylene oxide		IARC group 2B; REACH: carcinogenic, mutagenic
*N*-Acetyl-*S*-(3-hydroxypropyl)-L-cysteine	3HPMA		acrolein		IARC group 2 A
*N*-Acetyl-*S*-(2-cyanoethyl)-L-cysteine	2CyEMA		acrylonitrile		IARC group 1; REACH: carcinogenic, skin sensitizing
*N*-Acetyl-*S*-(*N*-methylcarbamoyl)-L-cysteine	MCaMA		N,N-dimethylformamide		IARC group 2 A, REACH: toxic to reproduction
*N*-Acetyl-*S*-(2-carbamoylethyl)-L-cysteine	2CaEMA		acrylamide		IARC group 2 A, REACH: carcinogenic, mutagenic, skin sensitizing
*N*-Acetyl-*S*-(2-carbamoyl-1-hydroxyethyl)-L-cysteine	2CaHEMA		glycidamide (metabolite of acrylamide)		REACH: carcinogenic, mutagenic, skin sensitizing
*N*-Acetyl-*S*-(3-hydroxy-1-methylpropyl)-L-cysteine	3HMPMA		crotonaldehyde		IARC group 2B, REACH: suspected to be mutagenic
*N*-Acetyl-*S*-(2-carboxy-1-methylethyl)-L-cysteine	2CoMEMA		crotonaldehyde		IARC group 2B, REACH: suspected to be mutagenic
*N*-Acetyl-*S*-(3,4-dihydroxybutyl)-L-cysteine	34HBMA		1,3-butadiene		IARC group 1, REACH: carcinogenic, mutagenic
*N*-Acetyl-*S*-(1-hydroxymethyl-2-propenyl)-L-cysteine	1HMPeMA		1,3-butadiene		IARC group 1, REACH: carcinogenic, mutagenic
*N*-Acetyl-*S*-(2-hydroxymethyl-3-buten-1-yl)-L-cysteine	2HBeMA		1,3-butadiene		IARC group 1, REACH: carcinogenic, mutagenic
*N*-Acetyl-*S*-(1-phenylethyl-2-hydroxy)-L-cysteine	2H1PhEMA		styrene		IARC group 2 A, REACH: suspected to be toxic to reproduction
*N*-Acetyl-*S*-(2-phenylethyl-2-hydroxy)-L-cysteine	2H2PhEMA		styrene		IARC group 2 A, REACH: suspected to be toxic to reproduction
*N*-Acetyl-*S*-phenyl-L-cysteine	PhMA		benzene		IARC group 1, REACH: carcinogenic, mutagenic
*N*-Acetyl-*S*-benzyl-L-cysteine	BzMA		toluene		IARC group 3, REACH: suspected to be toxic to reproduction

## Materials and methods

### Analytical method

18 MAs were determined by means of two LC-MS/MS methods as previously described [16]. The abbreviations for the MAs follow the harmonized acronym system introduced by Tevis et al. [17] (Table 1). One method applies a column-switching online extraction for the analysis of ten MAs in a 500 µL aliquot of urine (2-hydroxyethyl mercapturic acid (2HEMA), methyl mercapturic acid (MMA), ethyl mercapturic acid (EMA), 2-cyanoethyl mercapturic acid (2CyEMA), 3-hydroxy-1-methylpropyl mercapturic acid (3HMPMA), 2-carboxy-1-methylethyl mercapturic acid (2CoMEMA), 1-phenylethyl-2-hydroxy mercapturic acid (2H1PhEMA), 2-phenylethyl-2-hydroxy mercapturic acid (2H2PhEMA), phenyl mercapturic acid (PhMA), benzyl mercapturic acid (BzMA)). A second aliquot of 100 µL urine was completely evaporated and reconstituted in 100 µL methanol before analysis. Eight MAs were quantified for aliquot 2 (2-hydroxypropyl mercapturic acid (2HPMA), 3-hydroxypropyl mercapturic acid (3HPMA), 2-carbamoylethyl mercapturic acid (2CaEMA), 2-carbamoyl-2-hydroxy-ethyl mercapturic acid (2CaHEMA), methylcarbamoyl mercapturic acid (MCaMA), 1-hydroxymethyl-2-propenyl mercapturic acid (1HMPeMA), 2-hydroxymethyl-3-buten-1-yl mercapturic acid (2HBeMA), 3,4-dihydroxybutyl mercapturic acid (34HBMA)).

Sample analysis was conducted in accordance with ICH guideline M10 on bioanalytical method validation and study sample analysis [18]. The analytical runs were accepted based on the criteria for calibration and quality control (QC) samples. QC samples were interspersed throughout the run in three different concentration levels (low, medium, high) with a tolerance range of 85–115% accuracy. LLOQ ranged from 0.02 µg/L (SPMA) to 25 µg/L (3-HPMA).

### Study population

The biobanked aliquots of 24-h urine samples from the ESB collected in the years 2000, 2005, 2010, 2015, 2019, and 2021 were provided by the German Environment Agency (Umweltbundesamt, UBA). In total, 360 urine samples (60 per year with a 50/50 sex ratio) from non-occupationally exposed participants between 20 and 29 years of age from Münster (North Rhine-Westphalia, Germany) were analyzed in this study. The sampling location was chosen to facilitate the evaluation of time trends of exposure over more than 20 years and allowing for potential future mixture assessments based on measurements in the same samples. The study protocol of the ESB has been approved by the ethics committee of the Medical Association Westfalen-Lippe, the Medical Faculty of the University of Münster and (since 2012) by the ethical committee of the Medical Association of the Saarland. The study was conducted in compliance with both European and national legal and ethical requirements in accordance with the Declaration of Helsinki. All participants gave informed written consent to participate in the study. Information on total 24-h urine volume, urinary creatinine concentration, BMI, age and sex are summarized in Table 2. More information regarding sampling procedures and storage conditions of the ESB urine samples can be found elsewhere [19].

**Table 2:** Characteristics of the study population by sampling year and in total (2000–2021).

Year of sampling	N (m/f)	Age [years] mean	BMI [kg/m^2^] mean	Creatinine concentration [g/dL] mean	24-h urine volume [mL] mean
		(min-max)	(min-max)	(min-max)	(min-max)
2000	60 (30/30)	24.4	22.6	102	1665
		(20–29)	(15.4–30.5)	(30.6–203)	(800–4300)
2005	60 (30/30)	23.7	21.7	86.8	1906
		(20–28)	(17.0–27.0)	(18.9–241)	(721–3330)
2010	60 (30/30)	23.3	22.3	82.5	1927
		(20–28)	(18.0–29.3)	(27.1–219)	(790–3034)
2015	60 (30/30)	23.0	21.8	73.4	1937
		(20–28)	(16.6–27.1)	(21.6–220)	(560–2960)
2019	60 (30/30)	23.0	22.7	79.8	2018
		(20–28)	(18.0–34.6)	(19.9–223)	(738–3068)
2021	60 (30/30)	23.0	21.7	70.7	2142
		(20–28)	(16.9–27.8)	(26.2–171)	(1042–4918)
2000–2021	360 (180/180)	23.4	22.2	84.6	1932
		(20–29)	(15.4–34.6)	(18.9–241)	(560–4918)

### Statistical analysis

MA concentrations were normalized for 24-h urine volume and expressed as µg excreted in 24 h (µg/24 h). Values below LLOQ were set to LLOQ/2 for further analyses. Mean, geometric mean (GM), standard deviation (SD), median, and 95th percentile (P95) were calculated where appropriate. GM are discussed in the manuscript unless otherwise indicated. The data were tested for normal distribution by means of the D’Agostino-Pearson and Shapiro-Wilk tests showing that the MA concentrations were not normally distributed. Consequently, non-parametric Kruskal-Wallis ANOVA and post-hoc Dunn’s multiple comparisons test were performed to test for statistical significance (α = 0.05) regarding time-specific differences. Mann–Whitney U test was applied to verify sex-specific differences over all years and each specific year of collection (α = 0.05). To determine the effects of sex, smoking-status and BMI on MA levels, a multiple linear regression analysis was performed (*p* < 0.05; two-tailed). Due to the right-skewed distribution, the excreted amounts were log-transformed as outcome variables for each analyte. All statistical analyses were carried out using Prism 10.4.0 (GraphPad Software, LaJolla, CA, USA).

## Results

**Fig. 1:**
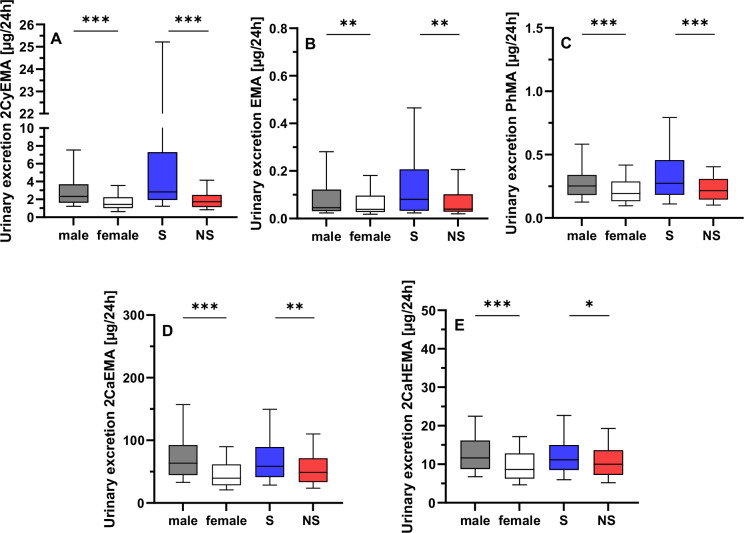
Daily urinary excretion (µg/24 h) of five mercapturic acids. Box plots for males in grey (*N* = 180), females in white (*N* = 180), smokers (S) in blue (*N* = 77) and non-smokers (NS) in red (N = 283) of 2CyEMA (**A**), EMA (**B**), PhMA (**C**), 2CaEMA (**D**), and 2CaHEMA (**E**). Boxes: 75th/25th percentile; whiskers: 90th/10th percentile; horizontal line: median. Significant differences between males / females and between S / NS, respectively, are illustrated by the asterisks (non-parametric Mann–Whitney U test). *: *p* < 0.05; **: *p* < 0.01; ***: *p* < 0.001.

No significant differences were observed in the study population for age, BMI, urinary creatinine, nor the 24-h urine volume (Table 2). The majority of the 24-h urine samples had quantifiable levels for most of the 18 MA analyzed: 2CoMEMA, 3HMPMA, 2HPMA, MCaMA, 34HBMA, 2CaHEMA (all 100%), PhMA, BzMA, 3HPMA (all 99%), 2CyEMA, 2HEMA, MMA (all 97%), 2CaEMA (95%). EMA, 2HBeMA, 2H1PhEMA, and 2H2PhEMA were found in 55%, 32%, 16%, and 5%, respectively, while 1HMPeMA was not detectable. With regard to the low detection rates, 2H1PhEMA, 2H2PhEMA, and 1HMPeMA were omitted from further analyses. The Spearman correlation matrix is illustrated in Figure S1 (Supplementary Information) for the 13 MAs with high detection rates (97-100%). A strong correlation between 2CaEMA and 2CaHEMA (r = 0.77) as well as between 2CoMEMA and 2HPMA (*r* = 0.67) was observed. Overall, MMA (0.07–0.59) and MCaMA (0.06–0.29) tended to show poor correlations (average *r* = 0.25 and 0.17) while the highest correlations were found for 2CyEMA, 2CaEMA, 2CaHEMA, and 2HPMA (average r = 0.42–0.44). Correlations for the 77 smokers (S), as well as for the 283 non-smokers (NS, based on self-report, and verified by urinary cotinine with a cut-off of 50 µg/L [20]), were very similar to the complete set of samples (cf. Supplementary Information Figure S2, S3). 2CyEMA, EMA, PhMA, 2CaEMA, and 2CaHEMA were significantly elevated in smokers (see Fig. 1, and Table 3) with 2CyEMA showing the most pronounced elevation of 2.4-fold, followed by EMA, SMPA and 2CaEMA/2CaHEMA with 1.7-, 1.4-, and 1.2-fold [16, 21]. We tested the time trends for 2CyEMA for all 360 urine samples (NS + S) as well as for the 283 NS and the 77 S separately (data not shown). Since no alterations in time trends and correlations were observed for 2CyEMA which represents the most significant difference between S and NS in these subpopulations compared to the complete study population, the data evaluation was performed for the whole sample set, independent of the smoking status.

**Table 3:** Descriptive statistics for urinary daily excretion (µg/24 h) of 15 MAs stratified by sex, smoking-status, and overall.

Sample (N)		2CyEMA***	2CoMEMA***	EMA**	2HEMA	3HMPMA***	MMA*	BzMA	PhMA***	2CaEMA***	2CaHEMA***	MCaMA	34HBMA***	2HPMA***	3HPMA***	2HBeMA
overall (360)	Mean	5.14	1406	0.138	1.78	190	24.1	16.8	0.284	63.1	11.8	151	674	29.2	424	0.391
	GM	2.13	1021	0.0609	1.36	159	18.6	12.8	0.224	51.7	10.3	119	620	25.2	300	0.201
	Median	1.91	1040	0.0430	1.32	157	17.9	12.7	0.226	52.0	10.3	122	635	24.6	281	0.151
	P95	11.4	3770	0.433	4.35	448	60.1	45.7	0.614	162	23.6	396	1158	63.5	1351	1.36
	Min-Max	0.272–380	101–8268	0.0110–2.72	0.188–23.1	16.0–896	2.88–203	0.244-153	0.0200-3.67	7.98-319	2.46-45.9	21.2-830	18.2-2574	6.39-144	30.7-5610	0.0470-6.73
female (180)	Mean	3.57	1030	0.0915	1.84	168	25.2	15.8	0.242	48.9	10.2	149	572	25.9	272	0.369
	GM	1.64	786	0.0510	1.37	141	19.9	12.2	0.196	41.1	8.95	116	540	21.9	227	0.198
	Median	1.44	785	0.0390	1.36	135	19.8	12.5	0.194	39.5	8.64	124	558	21.0	226	0.149
	P95	7.84	2806	0.254	4.68	409	66.1	43.6	0.499	117	21.9	400	904	64.7	625	1.60
	Min-Max	0.272–153	101–6343	0.0110–1.61	0.188–17.7	16.0–896	3.41–108	0.244-83.0	0.0200-2.13	7.98-185	2.79-45.4	21.2-483	140-1221	7.92-133	34.1-1722	0.0470-3.49
male (180)	Mean	6.71	1783	0.184	1.72	212	23.1	17.7	0.327	77.4	13.4	154	775	32.4	576	0.414
	GM	2.77	1327	0.0727	1.34	179	17.4	13.4	0.256	65.0	11.9	121	712	29.0	396	0.204
	Median	2.32	1262	0.0460	1.27	168	16.2	13.0	0.253	63.7	11.6	121	724	29.3	350	0.156
	P95	14.1	4697	1.09	3.53	504	56.8	47.7	0.692	185	28.9	385	1284	63.5	1878	1.26
	Min-Max	0.375–380	246–8268	0.0130–2.72	0.246–23.1	50.6–885	2.88–203	0.287-153	0.0250-3.67	12.3-319	2.46-45.9	22.6-830	18.2-2574	6.39-144	30.7-5610	0.0510-6.73
Smokers (77)	Mean	15.6	1475	0.233	2.17	204	26.1	17.8	0.426	73.7	13.3	164	677	30.1	431	0.395
	GM	4.20	1020	0.0914	1.51	168	19.3	13.1	0.292	61.5	11.6	128	637	25.9	313	0.201
	Median	2.84	981	0.0810	1.46	157	19.5	12.0	0.274	58.7	11.1	137	637	26.0	273	0.138
	P95	87.7	5562	1.48	6.92	574	74.7	51.5	1.49	165	31.9	398	1090	58.1	1400	1.38
	Min-Max	0.351–380	208–8268	0.0160–2.72	0.242–23.1	50.1–757	2.88–198	0.244-73.7	0.0250-3.67	12.3-319	2.70-45.9	25.8-830	121-1377	6.39-144	30.7-3105	0.0510-6.73
Non-smokers	Mean	2.30	1388	0.112	1.67	186	23.6	16.5	0.246	60.3	11.4	148	673	28.9	422	0.390
(283)	GM	1.77	1021	0.0545	1.32	157	18.4	12.7	0.208	49.3	9.99	116	616	25.1	296	0.201
	Median	1.74	1050	0.0400	1.30	157	16.9	12.7	0.216	48.9	10.0	120	635	24.3	281	0.155
	P95	5.90	3769	0.277	3.91	431	58.8	41.4	0.552	164	23.2	397	1190	64.8	1342	1.51
	Min-Max	0.272–21.8	101–6726	0.0110–2.54	0.188–18.3	16.0–896	3.18-203	0.287–153	0.0200-1.34	7.98-289	2.46-45.4	21.2-673	18.2-2574	7.92-133	34.1-5610	0.0470-6.73

Significant sex-specific differences are indicated by asterisks in the header for the respective MA (*: *p* < 0.05; **: *p* < 0.01; ***: *p* < 0.001; Mann–Whitney U test). For significant differences between smokers and non-smokers refer to Fig. 1.

*GM* geometric mean; *P95* 95th percentile, *Min* minimum, *Max* maximum.

GMs ranged between 1021 µg/24 h (567 µg/L) for 2CoMEMA and 0.0609 µg/24 h (0.0338 µg/L) for EMA in the following order (see Table 3): 2CoMEMA > 34HBMA > 3HPMA > 3HMPMA > MCaMA > 2CaEMA > 2HPMA > MMA > BzMA > 2CaHEMA > 2CyEMA > 2HEMA > PhMA > 2HBeMA > EMA.

Urinary excretions (µg/24 h) were investigated across all years and for each sampling year and sex to determine time trends and sex-specific differences. Urinary concentrations (µg/L) and creatinine-normalized values (µg/g creatinine) are summarized in Table S1 and S2, respectively (Supplementary Information). BzMA was the only analyte with high detection rates (99%) with neither time nor sex-specific alterations. 2HBeMA also showed no differences in the statistical analysis, but with a much lower detection rate of 32%.

11 MAs showed a significant difference between males and females (Table 3). Males had higher levels in 10 of these 11 MAs (2CyEMA (Fig. 1A), 2CoMEMA, 3HMPMA, EMA (Fig. 1B), PhMA (Fig. 1C), 2CaEMA (Fig. 1D), 2CaHEMA (Fig. 1E), 34HPMA, 2HPMA, and 3HPMA), while MMA was the only analyte with a significantly higher excretion in females. This difference was significant across all years and for most of the individual collection years, except for MMA, where the sex-specific difference was only significant when comparing the complete study population (2000–2021, *N* = 360). The highest sex-specific differences occurred for 2CyEMA (1.7-fold) and 2CaEMA (1.6-fold), while the other MAs were in the range of 1.3 to 1.4-fold higher in males compared to females. A separate evaluation of S and NS revealed the same differences (data not shown).

In addition, we performed multiple linear regression including sex, smoking-status, and BMI to account for the contribution of these covariables. Sex and smoking contribute to statistically significant differences for the same MAs as found in the univariate analysis (Table S3), except for EMA which did not show a sex-specific difference in the multiple linear regression. The most profound increase in men was identified for 3HPMA with an elevation by 40% compared to women, while smoking status and BMI did not affect 3HPMA levels. Moreover, the BMI significantly impacts the MA levels for 14 MAs (2CyEMA, 2CoMEMA, EMA, 2HEMA, 3HMPMA, MMA, BzMA, PhMA, 2CaEMA, 2CaHEMA, MCaMA, 34HBMA, 2HPMA, and 2HBeMA) while no significant difference was obtained for 3HPMA. The overall variance which can be explained by these covariables differs substantially by MA as expressed by R^2^ in Table S3 (variance in MA level attributable to MA concentrations = R^2^ x 100 in percent). The investigated covariables contributed to a larger extent to the overall amount of 2CyEMA, 2CoMEMA, 2CaEMA, 2CaHEMA, 34HBMA, and 3HPMA in the range of 24% (2CyEMA) to 15%. The percentage change in MA levels for one covariable can be calculated from the respective regression coefficient (β) [22]. With each unit change in the covariable, the outcome (MA excretion) changes by 100*[exp (β)-1] (%). The smoking status is the most relevant predictor in terms of 2CyEMA, EMA and PhMA with 27 to 56% change. For the other MAs, sex is primarily driving the differences in MA excretion, whereas BMI, while showing a significant correlation, only provides a minor contribution to the changes in MA levels in the range of 2–9% change with increasing BMI.

**Fig. 2:**
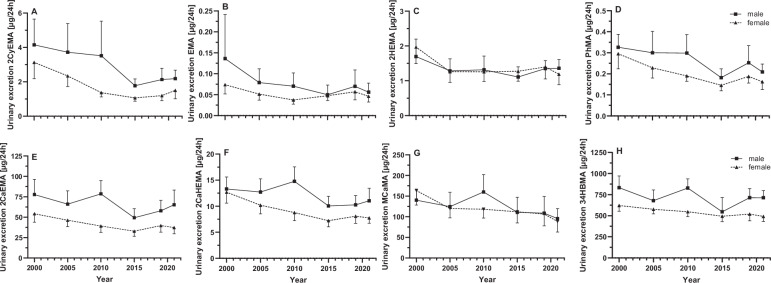
Time course of the urinary excretion of eight MAs. Urinary excretions (µg/24 h) depicted as GM ± 95% CI of 2CyEMA (**A**), EMA (**B**), 2HEMA (**C**), PhMA (**D**), 2CaEMA (**E**), 2CaHEMA (**F**), MCaMA (**G**), 34HBMA (**H**) in males (*N* = 30 per year of collection) and females (*N* = 30 per year of collection). All MAs illustrated here showed statistically significant decrease (Kruskal-Wallis ANOVA; *p* < 0.05).

A significant decrease over time (Kruskal Wallis ANOVA, *p* < 0.05) was observed for 8 MAs (2CyEMA, EMA, 2HEMA, PhMA, 2CaEMA, 2CaHEMA, MCaMA, 34HBMA) with 18 to 50% reductions from 2000 to 2021 (Fig. 2). Differentiated by sex, the same time trend was obtained for 2CyEMA, PhMA, 2CaEMA, 2CaHEMA for both sexes and additionally for MCaMA and 34HBMA for the male population. The time trend for EMA and 2HEMA was only significant for the complete population under scrutiny. Dunn’s test for multiple comparisons revealed that the decrease was in general most pronounced in 2015 compared to previous collection years, while the downward trend leveled off in the following years 2019 / 2021. For instance, 2CyEMA, showing the strongest overall reduction of 50%, had a steep decline between 2010 and 2015, with no further decrease in 2019. As an exception, MCaMA showed a significant decrease only in 2021 compared to the first collection year in 2000. A few MAs appear to have a higher excretion rate in 2010 for the male samples (2CaEMA, 2CaHEMA, MCaMA, 34HBMA, 3HPMA), which did not reach statistical significance.

## Discussion

### General considerations and detection rates

The MA pathway is an important detoxification mechanism in the body that results in the excretion of the corresponding VOC metabolites in urine [13]. In general, the correlation between the different MAs was moderate to weak with high significance (*p* < 0.001). This suggests different exposure sources such as diet, tobacco smoke, or ambient air. Comprehensive biomonitoring data related to VOC exposure in larger European cohorts are scarce. PhMA and 2CaEMA/2CaHEMA are the only MAs for which HBM data in a population-representative sample in Germany have been assessed so far in children within GerES V [23]. PhMA averaged 0.097 µg/L (GM) with 95th percentile (P95) of 0.41 µg/L which is slightly below the mean PhMA concentration in our study of 0.124 µg/L with a comparable P95 of 0.42 µg/L, despite the far lower proportion of smokers in GerES V (2%) compared to the current HBM assessment in ESB samples (21%). This indicates the importance of other sources than smoking, like oil heating and air traffic pollution which were positively correlated with PhMA [23]. An HBM study conducted in Italy in 2013–2014 (adults aged 35–69; *N* = 1076) reported similar PhMA concentrations of 0.139 µg/g creatinine (GM of 0.167 µg/g creatinine in our study) [24]. Acrylamide exposure was around 2.5-times higher in GerES V with 72.6 µg/L 2CaEMA and 15.0 µg/L 2CaHEMA. Consumption of fried food was identified as the major contributor to elevated 2CaEMA concentrations besides smoking. In contrast, comparable levels were found in 20-40 year old adults in Poland (median 2CaEMA: 20.9 µg/L vs 28.5 µg/L; median 2CaHEMA: 8.6 µg/L vs 5.95 µg/L) at a similar proportion of smokers of 16% compared to our study [25].

Given the data gap in Germany and Europe in general related to VOC exposure in adults, the following discussion will refer to data from the NHANES study of the adult (20+ years) U.S. population covering the years 2005 to 2018 [14].

In total, 14 out of 18 MAs were found at very high detection rates of 95% and more, proving their applicability in the HBM of VOCs and providing the first baseline concentration levels for a large number of MAs in non-occupationally exposed young adults in Germany. For BzMA, 3HPMA and 34HBMA, the median concentrations are comparable with NHANES data for the respective time span (Table 4). In contrast, 2CyEMA, 3HMPMA, 2CaEMA, MCaMA, and 2HPMA, appear to be less concentrated in the ESB samples. We confirmed this observation using creatinine-normalized values (data not shown) to rule out dilution effects as a cause for these differences. However, the comparability may be limited owing the difference in the study populations, where NHANES is population-representative in contrast to the 24-h urine samples from 20- to 29-year-old students in the ESB. Some MAs (2HEMA, PhMA, 2CaHEMA) were not detectable in NHANES, which reported LODs above the median concentrations found in the ESB samples. Obviously, a higher sensitivity is required for an HBM in the general population, which may be compromised in the analysis applied by the CDC in favor of a broader range of analytes. For comparison, our analysis comprises 18 MAs measured by two separate methods, while the HBM program for VOCs in NHANES determines 28 urinary VOC metabolites in one run [21].

**Table 4:** Comparison of the median concentrations in our study versus NHANES (µg/L).

MA	Study	2000	2005/2006	2010	2011/2012	2013/2014	2015/2016	2017/2018	2019	2021
2CyEMA	present study (ESB)	1.96	1.25	1.14			0.825		0.824	0.863
	NHANES		1.88		1.78	1.69	1.48	1.44		
2HEMA	present study (ESB)	1.22	0.661	0.665			0.699		0.727	0.568
	NHANES		<LOD^a^		0.895	<LOD^a^	<LOD^a^	<LOD^a^		
3HMPMA	present study (ESB)	119	97.5	84.3			75.8		86.0	75.9
	NHANES		231		248	222	248	214		
BzMA	present study (ESB)	10.5	6.97	6.90			6.17		6.42	6.01
	NHANES		6.62		6.31	6.24	6.16	5.75		
PhMA	present study (ESB)	0.240	0.135	0.141			0.0965		0.125	0.0910
	NHANES		<LOD^b^		<LOD^b^	<LOD^b^	<LOD^b^			
2CaEMA	present study (ESB)	42.3	31.4	27.9			21.2		26.4	24.2
	NHANES		56.3		45.2	45.8	51.6	51.8		
2CaHEMA	present study (ESB)	8.30	6.45				6.55		5.45	4.90
	NHANES		11.8		<LOD^c^	<LOD^c^	<LOD^c^			
MCaMA	present study (ESB)	104	68.9	67.5			58.4		54.9	48.4
	NHANES		150		163	141	145	157		
34HBMA	present study (ESB)	484	382	392			296		328	293
	NHANES		294		281	267	348	350		
2HPMA	present study (ESB)	17.4	13.8	13.3			11.9		15.5	12.0
	NHANES		37.4		32.1	25.3	30.6	29.0		
3HPMA	present study (ESB)	204	152	163			140		144	138
	NHANES		247		220	210	249	246		

^a^LOD = 0.791 µg/L.

^b^LOD = 0.600 µg/L.

^c^LOD = 9.40 µg/L.

Focusing on behavioral aspects, smokers had higher levels of 5 MAs compared to non-smokers deriving from acrylonitrile (2CyEMA), ethylating agents (EMA), benzene (PhMA), and acrylamide (2CaEMA/2CaHEMA). These differences have been reported previously by several groups [16, 21, 26–28]. In addition, the US Population Assessment of Tobacco and Health (PATH), the most comprehensive population-based longitudinal study of tobacco use worldwide, confirmed our findings for the respective MAs [21, 29]. Yet, several MAs such as 2-HPMA, 3-HPMA, or MCaMA did not show a significant difference in our study, which is in contrast to the findings of the PATH and other studies [21, 29, 30]. The low number of 77 smokers and the concise classification by self-report (yes/no) and cotinine levels without detailed stratification by cigarettes per day or smoking history for the ESB samples may result in the misclassification. This applies in particular to vapers or users of other nicotine products, who have high cotinine levels but no pronounced exposure to the precursors of the respective tobacco smoke constituents propylene oxide, acrolein, or dimethylformamide. In this context it has to be noted that the discrimination between S and NS was not the primary scope in this study.

2H1PhEMA and 2H2PhEMA, both biomarkers of styrene exposure, were rarely detected, which is consistent with NHANES study data in the U.S. population [14]. This indicates the need for alternative biomarkers to monitor styrene exposure. While both MAs are specific to styrene, they lack in sensitivity due to the low excretion rate of only 1% of the styrene dose [31]. Mandelic acid (hydroxy(phenyl)acetic acid) remains the primary choice as a more sensitive biomarker for styrene exposure, although it is less specific [32].

1,3-BD forms at least four MAs [33], with 2HBeMA and 34HBMA being most frequently applied in biomonitoring studies [34]. In contrast, 1HMPeMA is a minor metabolite not detectable in the general population as shown in the present study and in NHANES [14]. Only one study has measured 1,3-BD exposure on a population-representative scale in 5897 subjects for NHANES cycles between 2011 and 2016 and reported 34HBMA (25th to 75th percentile (P25-P75): 211–465 µg/g creatinine) in more than 96% and 2HBeMA in 9.8% of the samples, respectively [35]. Our study had somewhat higher detection rates for 34HBMA (100%) and 2HBeMA (32%) with a comparable concentration range of 380-564 µg/g creatinine for 34HBMA (P25-P75).

### Sex-specific differences in MA excretion

The main aim of the study was to decipher potential differences between sexes and the overall exposure over time. Males had higher urinary excretions for 10 of the 18 MAs which are related to the exposure to acrylonitrile (2CyEMA), crotonaldehyde (2CoMEMA/3HMPMA), ethylating agents (EMA), benzene (PhMA), acrylamide (2CaEMA/2CaHEMA), 1,3-BD (34HBMA), propylene oxide (2HPMA), and acrolein (3HPMA). Interestingly, MMA was the only analyte with significantly higher levels in females while EMA follows the trend found for the 9 MAs listed above. The assessment of MMA and EMA has not been reported in large scale studies so far. MMA and EMA are indicative of the exposure to toxic methylating and ethylating agents such as N-nitrosodimethylamine, N-nitrosodiethylamine, methyl chloride or ethyl chloride [36].

The comparison of urinary VOC metabolites between males and females has been previously reported in only two larger studies, both evaluating the data from the US NHANES 2011/2012 cycle, albeit in different subpopulations [37, 38]. Jain (2015) compared the adjusted GMs separated by sex and smoking status [37] while Mendy et al. (2022) used the creatinine-normalized values in a subset for which spirometry data were available [38]. Mendy et al. (2022) reported elevated levels of 3HMPMA and 2HPMA in females, which contradicts to our findings. In contrast, Jain (2015) found higher 3HPMA concentrations in female non-smokers in agreement with our results. However, the evaluation by Jain did not observe any sex-specific differences in non-smokers for 1,3-BD and crotonaldehyde. Hartmann et al. (2008) identified differences in 2CaEMA and 2CaHEMA with 1.5-fold higher urinary concentrations in men, similar to our study (1.6-fold elevation in men), while this sex difference disappeared for the creatinine-based concentrations [39]. It is important to note that women generally have lower creatinine concentrations due to differences in physiology and metabolism [40]. This would suggest that the concentration in females may be higher after normalization, even if there is no actual difference in exposure or a similar exposure, although men could be more exposed. In our study, concentrations were adjusted for the 24-h urine volume, which should not be influenced by additional factors like body weight, age, or muscle mass [41]. It is noteworthy that sex-specific differences were observed in both the complete study population as well as for the smokers and non-smokers individually. Variations in VOC exposures have been discussed in relation to the physiological differences between sexes in terms of absorption, deposition, and metabolism (ADME) of environmental chemicals [42]. For instance, sex hormones can influence GI tract motility, potentially leading to the accumulation of the ingested chemicals before excretion, which is especially pertinent for VOCs absorbed through dietary intake [43]. Regarding inhalation, which is the primary route of exposure for most VOCs, differences in ventilation capacity, tidal volumes, and lung size may result in varying uptakes between sexes [42]. However, it is likely that other exogenous sources, in addition to active smoking of combustible cigarettes and/or differences in the ADME of VOCs, likely contribute to the observed differences. For instance, diet significantly contributes to acrylamide exposure as reflected by higher CaEMA/CaHEMA levels after consumption of fried potatoes [44]. Moreover, various fruits contain crotonaldehyde and their consumption was linked to elevated 3HMPMA levels [45]. For most VOCs, their primary source can be attributed to the ubiquitous presence in ambient air, stemming from industrial and traffic emissions. Disparities in exposure may be linked to differences in occupational settings or residence. However, most studies have not found a causal relationship between sex and exposure levels [23, 44]. The observed sex- and smoking-specific differences illustrate the potential of HBM data to improve our understanding of the main sources of exposure to this highly toxic class of chemicals.

### Influence of different covariables on MA levels

Our multiple linear regression model included three covariables: sex, smoking status and BMI. As discussed in the previous section 4.2, sex-specific differences may be attributed to differences in the ADME of VOCs as well as lifestyle and use behavior. However, the manifold environmental sources of exposure are too heterogenous to provide a simple answer for the observed variance. One significant contributor is the BMI, which correlates with dietary habits [46]. Our study suggests that BMI is positively correlated with the excreted amounts of MA coming from acrylonitrile, crotonaldehyde, ethylene oxide, toluene, benzene, acrylamide, dimethylformamide, 1,3-butadiene, propylene oxide, and alkylating agents, in agreement with previous reports [47, 48]. A more detailed investigation regarding dietary effects in a European cohort, similar to Lei et al [47], requires a comprehensive questionnaire, as available for instance in the GerES. In general, smoking contributes to a much higher extent to the exposure to several VOCs like acrylonitrile, benzene or acrylamide with an increase in MA levels of 56%, 27%, and 17% for 2CyEMA, PhMA, and 2CaEMA, respectively.

### Time trends in VOC exposure from 2000 to 2021

In the present study, a notable reduction in MA concentrations was observed from 2000 to 2021, with significant decreases for 2CyEMA (50%), EMA (50%), 2HEMA (31%), PhMA (41%), 2CaEMA (24%), 2CaHEMA (29%), MCaMA (39%), and 34HBMA (18%), irrespective of sex and smoking status. We want to draw attention to the fact that while several classes of environmental chemicals, such as metals, PFAS, phthalates and PAHs have been extensively studied in terms of changes in exposure over time [15, 49–52], the assessment of MA VOC metabolites in HBM studies in this context is still limited. Poteser et al. performed a comprehensive analysis of acrylamide HBM data across ten European countries revealing an overall increase from 2001 to 2017 followed by a decline starting in 2018 in adults [53]. Notably, benchmark levels in food were adopted in 2017 which came into effect early 2018 in Europe may have influenced this trend [53]. In contrast, our data for 2CaEMA/2CaHEMA show a different time trend with the lowest value and most significant drop in 2015 and no significant increase in the years before.

In the population-representative Canadian study (CHMS) within 6 years from 2009-2015 [54], a significant decline in PhMA (18%) was observed, similar to our findings. Moreover, a substantial decline in acrylonitrile (2CyEMA) and N,N-dimethylformamide (MCaMA) exposure was observed in an extensive evaluation of NHANES data from 2012 onwards (Table 4), which falls within the time frame of the most pronounced decline in our study [55]. With regard to the major sources and given the fact that the decrease is independent of the smoking status, a reduction in secondhand smoke exposure seems a probable cause for the observed trends. Secondhand smoke exposure has steadily decreased in Germany since 2002 as a result of smoking bans and regulations limiting secondhand smoke exposure [20]. Moreover, the exposure to polycyclic aromatic hydrocarbons showed a similar time trend, which was attributed to reduced secondhand smoke exposure and legislation that has been implemented in the EU over the past 20 years to minimize chemical emissions into the air and improve air quality [20]. Hence, besides secondhand smoke exposure, environmental air and diet may contribute to the reduced exposure to these VOCs. Yet, closing data gaps and establishing a causal relationship requires comprehensive and regular monitoring of VOCs. This emphasizes the importance of national representative surveys such as GerES to facilitate the correlation of MA levels with the participants’ habits, diet, and socioeconomic status.

### Exposure assessment

Since many of the analyzed VOC metabolites have parent compounds which are carcinogens, mutagens or toxic for reproduction (see Table 1) deriving a safe level of exposure is difficult and only few health-based guidance values are available.

All urine samples in this study showed MCaMA concentrations below the HBM guidance value (HBM-GV) of 1000 µg/g creatinine [56], indicating a low health-risk from N,N-dimethylformamide exposure. For non-cancer toxicity, Hays and Aylward derived a Biomonitoring Equivalent (BE) value consistent with a health protective guidance value for the general population for 2CaEMA of 13 μg/L urine [57]. For cancer risk, they calculated a BE value for urinary 2CaEMA concentrations of 1 μg/L (for a risk of 1 × 10^–4^) and of 0.01 μg/L (for a risk of 1 × 10^–6^). Most urinary 2CaEMA concentrations found in this study (with an LLOQ of 10 µg/L for 2CaEMA; 95% > LLOQ) were higher than the BE values. Therefore, individual avoidance as well as further regulation are necessary to reduce hazardous exposure, which holds true for all VOCs.

## Conclusion

This is the first comprehensive HBM of a large number of VOCs in a non-occupationally exposed population based on the respective MA metabolites in a European country. The analysis of 360 24-h urine samples from the ESB spanning 21 years showed widespread detection of 14 MAs, indicative of their applicability in HBM. The multivariate analysis identified BMI and smoking-status as confounders for several MA metabolites. Moreover, significant differences between males and females underscore the importance of considering sex-specific exposure patterns. The high detection rates, sex-specific differences and time trends emphasize the need for a closely meshed HBM, preferably in the German Environmental Survey, in order to fill existing data gaps, establish reference values, and further elucidate the sources and health implications of VOC exposure. HBM derived reference values will improve our understanding of the health consequences from the exposure of the general population to VOCs, as exemplified for the guidance-value based exposure assessment of DMF and acrylamide.

## Supplementary information

Supplementary information

## Data Availability

Additional data are available from the corresponding author on reasonable request.
