# Supplementary material for: Analysis of 18 mercapturic acids in urine samples from the German Environmental Specimen Bank—tackling the data gap in the human biomonitoring of VOCs in Europe
Source: J Expo Sci Environ Epidemiol. 2026 Jan 28;36(3):490–503. doi: 10.1038/s41370-026-00838-x (PMC13143828; doi:10.1038/s41370-026-00838-x)
Supplement: Supplementary file 1 — Supplementary information [file 41370_2026_838_MOESM1_ESM.docx]

**Supplementary Information to:**

**Analysis 18 mercapturic acids in urine samples from the German Environmental Specimen Bank – Tackling the data gap in the human biomonitoring of VOCs in Europe**

Nikola Pluym^a^, Therese Burkhardt^a^, Till Weber^b^, Gerhard Scherer^a^, Max Scherer^a^*, Marike Kolossa-Gehring^b^

^a^ ABF Analytisch-Biologisches Forschungslabor GmbH, Semmelweisstr. 5, 82152 Planegg, Germany
^b^ German Environment Agency (UBA), Corrensplatz 1, 14195 Berlin, Germany

^*^ Corresponding author, e-mail address: max.scherer@abf-lab.com (M. Scherer).

Table S1: Descriptive statistics for urinary concentrations (µg/L) of 15 MAs stratified by sex and overall.

| Sample (N) |  | 2CyEMA | 2CoMEMA | EMA | 2HEMA | 3HMPMA | MMA | BzMA | PhMA | 2CaEMA | 2CaHEMA | MCaMA | 34HBMA | 2HPMA | 3HPMA | 2HBeMA |
| --- | --- | --- | --- | --- | --- | --- | --- | --- | --- | --- | --- | --- | --- | --- | --- | --- |
| overall (360) | Mean | 3.18 | 795 | 0.078 | 1.04 | 110 | 14.1 | 9.82 | 0.171 | 37.7 | 6.99 | 88.7 | 405 | 16.9 | 257 | 0.209 |
|  | GM | 1.18 | 567 | 0.0338 | 0.753 | 88.3 | 10.3 | 7.1 | 0.124 | 28.7 | 5.73 | 65.9 | 344 | 14.0 | 166 | 0.112 |
|  | Median | 1.07 | 551 | <LLOQ | 0.723 | 91.4 | 10.3 | 6.86 | 0.125 | 28.5 | 5.95 | 66.2 | 344 | 13.7 | 155 | <LLOQ |
|  | P95 | 7.56 | 2389 | 0.305 | 2.96 | 254 | 38.5 | 27.3 | 0.423 | 97.8 | 15.5 | 225 | 832 | 36.8 | 739 | 0.696 |
|  | Min-Max | <LLOQ-298 | 36.9-5060 | <LLOQ-1.60 | <LLOQ-18.1 | 5.85-567 | <LLOQ-92.6 | <LLOQ-88.3 | <LLOQ-2.88 | <LLOQ-250 | <LLOQ-38.1 | 7.30-651 | <LLOQ-2860 | 2.60-113 | <LLOQ-4400 | <LLOQ-4.64 |
| female (180) | Mean | 2.00 | 604 | 0.0506 | 1.09 | 99.0 | 15.2 | 9.49 | 0.146 | 30.0 | 6.30 | 89.0 | 349 | 15.2 | 167 | 0.205 |
|  | GM | 0.922 | 443 | 0.0287 | 0.772 | 79.3 | 11.2 | 6.88 | 0.111 | 23.2 | 5.04 | 65.4 | 304 | 12.4 | 128 | 0.112 |
|  | Median | 0.836 | 459 | <LLOQ | 0.756 | 82.8 | 11.3 | 6.44 | 0.110 | 22.3 | 5.20 | 66.7 | 297 | 11.7 | 127 | <LLOQ |
|  | P95 | 6.42 | 1539 | 0.144 | 3.3 | 229 | 39.9 | 24.8 | 0.388 | 88.4 | 15.5 | 239 | 705 | 37.7 | 374 | 0.716 |
|  | Min-Max | <LLOQ-61.2 | 36.9-3040 | <LLOQ-0.739 | <LLOQ-6.30 | 5.85-475 | <LLOQ-86.7 | <LLOQ-88.3 | <LLOQ-1.49 | <LLOQ-154 | 1.20-32.7 | 8.10-523 | 49.8-940 | 2.90-85.1 | <LLOQ-1190 | <LLOQ-2.33 |
| male (180) | Mean | 4.36 | 986 | 0.105 | 0.995 | 120 | 12.9 | 10.2 | 0.196 | 45.4 | 7.68 | 88.5 | 461 | 18.7 | 347 | 0.213 |
|  | GM | 1.51 | 726 | 0.0398 | 0.734 | 98.2 | 9.52 | 7.33 | 0.140 | 35.6 | 6.51 | 66.4 | 390 | 15.9 | 217 | 0.111 |
|  | Median | 1.31 | 654 | 0.0315 | 0.659 | 97.2 | 9.39 | 7.13 | 0.138 | 35.7 | 6.55 | 65.8 | 406 | 15.4 | 189 | <LLOQ |
|  | P95 | 10.5 | 2843 | 0.539 | 2.34 | 306 | 34.3 | 28.5 | 0.497 | 104 | 15.8 | 213 | 948 | 36.4 | 1130 | 0.696 |
|  | Min-Max | <LLOQ-298 | 100-5060 | <LLOQ-1.60 | <LLOQ-18.1 | 16.5-567 | <LLOQ-92.6 | <LLOQ-71.6 | <LLOQ-2.88 | <LLOQ-250 | <LLOQ-38.1 | 7.30-651 | <LLOQ-2860 | 2.60-113 | <LLOQ-4400 | <LLOQ-4.64 |
| GM: geometric mean; P95: 95th percentile; Min: minimum; Max: maximum. | | | | | | | | | | | | | | | | |

Table S2: Descriptive statistics for creatinine-normalized urinary concentrations (µg/g creatinine) of 15 MAs stratified by sex and overall.

| Sample (N) |  | 2CyEMA | 2CoMEMA | EMA | 2HEMA | 3HMPMA | MMA | BzMA | PhMA | 2CaEMA | 2CaHEMA | MCaMA | 34HBMA | 2HPMA | 3HPMA | 2HBeMA |
| --- | --- | --- | --- | --- | --- | --- | --- | --- | --- | --- | --- | --- | --- | --- | --- | --- |
| overall (360) | Mean | 3.25 | 989 | 0.0939 | 1.30 | 139 | 18.2 | 12.4 | 0.203 | 44.3 | 8.40 | 114 | 489 | 21.4 | 303 | 0.296 |
|  | GM | 1.59 | 762 | 0.0454 | 1.01 | 119 | 13.9 | 9.54 | 0.167 | 38.6 | 7.70 | 88.6 | 463 | 18.8 | 224 | 0.150 |
|  | Median | 1.40 | 740 | 0.0382 | 1.01 | 114 | 13.2 | 9.41 | 0.163 | 37.2 | 7.71 | 90.5 | 477 | 18.1 | 205 | 0.119 |
|  | P95 | 7.66 | 2484 | 0.300 | 3.09 | 307 | 49.8 | 32.8 | 0.440 | 95.0 | 16.1 | 296 | 775 | 46.2 | 894 | 1.17 |
|  | Min-Max | 0.243-158 | 171-6118 | 0.00683-1.65 | 0.157-13.9 | 27.1-851 | 1.61-126 | 0.152-69.4 | 0.0191-1.92 | 8.19-148 | 1.89-29.4 | 18.3-487 | 10.0-1466 | 6.35-149 | 20.0-3192 | 0.0268-3.97 |
| female (180) | Mean | 2.95 | 933 | 0.0849 | 1.62 | 154 | 22.9 | 14.3 | 0.217 | 43.2 | 8.93 | 138 | 518 | 23.5 | 254 | 0.343 |
|  | GM | 1.50 | 720 | 0.0467 | 1.26 | 129 | 18.3 | 11.2 | 0.180 | 37.7 | 8.20 | 106 | 495 | 20.1 | 208 | 0.182 |
|  | Median | 1.39 | 723 | 0.0387 | 1.25 | 122 | 19.2 | 11.9 | 0.177 | 36.5 | 8.15 | 106 | 508 | 19.2 | 201 | 0.137 |
|  | P95 | 6.37 | 2237 | 0.268 | 4.07 | 365 | 58.9 | 36.8 | 0.444 | 91.1 | 16.1 | 349 | 814 | 54.1 | 531 | 1.33 |
|  | Min-Max | 0.243-115 | 171-6118 | 0.00907-1.57 | 0.157-13.9 | 27.1-851 | 3.15-79.9 | 0.214-69.4 | 0.0191-1.92 | 8.19-140 | 3.01-29.4 | 18.3-487 | 150-946 | 6.35-149 | 57.8-2594 | 0.0317-3.30 |
| male (180) | Mean | 3.54 | 1046 | 0.103 | 0.985 | 125 | 13.6 | 10.4 | 0.189 | 45.4 | 7.88 | 90.8 | 459 | 19.3 | 351 | 0.248 |
|  | GM | 1.68 | 805 | 0.0442 | 0.815 | 109 | 10.6 | 8.14 | 0.155 | 39.5 | 7.23 | 73.7 | 432 | 17.6 | 240 | 0.124 |
|  | Median | 1.42 | 799 | 0.0355 | 0.796 | 106 | 9.91 | 8.25 | 0.151 | 37.7 | 7.11 | 70.4 | 442 | 17.4 | 212 | 0.0925 |
|  | P95 | 7.79 | 2738 | 0.496 | 2.02 | 288 | 32.4 | 25.3 | 0.440 | 98.1 | 15.5 | 224 | 690 | 36.9 | 1210 | 0.850 |
|  | Min-Max | 0.324-158 | 177-4275 | 0.00683-1.65 | 0.167-9.62 | 38.4-443 | 1.61-126 | 0.152-62.3 | 0.0293-1.53 | 8.34-148 | 1.89-23.5 | 20.8-346 | 10.0-1466 | 6.40-60.1 | 20.0-3192 | 0.0268-3.97 |
| GM: geometric mean; P95: 95th percentile; Min: minimum; Max: maximum. | | | | | | | | | | | | | | | | |

Table S3: Results of the multiple linear regression analysis of 15 MAs. Significant differences by covariable highlighted in bold (p < 0.05).

| Characteristic | 2CyEMA | 2CoMEMA | EMA | 2HEMA | 3HMPMA | MMA | BzMA | PhMA | 2CaEMA | 2CaHEMA | MCaMA | 34HBMA | 2HPMA | 3HPMA | 2HBeMA |
| --- | --- | --- | --- | --- | --- | --- | --- | --- | --- | --- | --- | --- | --- | --- | --- |
| R^2^ | 0.244 | 0.149 | 0.102 | 0.041 | 0.056 | 0.046 | 0.019 | 0.102 | 0.183 | 0.148 | 0.057 | 0.146 | 0.117 | 0.147 | 0.012 |
| β0: Intercept | 0.356 | 5.587 | -4.163 | -0.793 | 4.638 | 1.613 | 1.719 | -1.976 | 3.228 | 1.409 | 3.306 | 5.683 | 2.337 | 5.540 | -2.620 |
| β1: BMI | **0.056** | **0.067** | **0.082** | **0.051** | **0.025** | **0.055** | **0.038** | **0.037** | **0.047** | **0.050** | **0.067** | **0.038** | **0.045** | 0.020 | **0.044** |
| %-change BMI | **5.7%** | **7.0%** | **8.6%** | **5.2%** | **2.6%** | **5.6%** | **3.9%** | **3.8%** | **4.8%** | **5.1%** | **6.9%** | **3.9%** | **4.6%** | 2.0% | **4.5%** |
| p-value BMI | **0.002** | **0.000** | **0.000** | **0.001** | **0.041** | **0.000** | **0.019** | **0.007** | **0.000** | **0.000** | **0.000** | **0.000** | **0.000** | 0.183 | **0.041** |
| β2: Sex (female) | **-0.381** | **-0.378** | -0.161 | 0.135 | **-0.186** | **0.255** | -0.010 | **-0.177** | **-0.351** | **-0.173** | 0.102 | **-0.193** | **-0.182** | **-0.513** | 0.069 |
| %-change Sex (female) | **-31.7%** | **-31.5%** | -14.9% | 14.5% | **-17.0%** | **29.1%** | -1.0% | **-16.2%** | **-29.6%** | **-15.9%** | 10.8% | **-17.5%** | **-16.7%** | **-40.1%** | 7.1% |
| p-value Sex (female) | **0.000** | **0.000** | 0.166 | 0.079 | **0.005** | **0.001** | 0.910 | **0.015** | **0.000** | **0.002** | 0.200 | **0.000** | **0.001** | **0.000** | 0.544 |
| β3: Smoking status (NS) | **-0.825** | 0.045 | **-0.474** | -0.123 | -0.052 | -0.031 | -0.012 | **-0.314** | **-0.187** | **-0.125** | -0.072 | -0.009 | -0.007 | -0.027 | 0.015 |
| %-change Smoking status (NS) | **-56.2%** | 4.6% | **-37.8%** | -11.6% | -5.0% | -3.1% | -1.2% | **-27.0%** | **-17.1%** | **-11.7%** | -6.9% | -0.9% | -0.7% | -2.6% | 1.5% |
| p-value Smoking status (NS) | **0.000** | 0.638 | **0.000** | 0.152 | 0.484 | 0.724 | 0.897 | **0.000** | **0.011** | **0.042** | 0.418 | 0.858 | 0.912 | 0.762 | 0.906 |
| BMI: Body mass index; NS: non-smoker | | | | | | | | | | | | | | | |

Figure S1: Spearman correlation matrix for the 13 MAs with high detection rates (N = 360).

0.00

0.00

Figure S2: Spearman correlation matrix for the 13 MAs with high detection rates in smokers (N = 77).

Figure S3: Spearman correlation matrix for the 13 MAs with high detection rates in non-smokers (N = 283).
